# Vitamin D_3_ alleviates cognitive impairment through regulating inflammatory stress in db/db mice

**DOI:** 10.1002/fsn3.2397

**Published:** 2021-07-07

**Authors:** Xiaomu Tan, Lifang Gao, Xiaxia Cai, Mingyuan Zhang, Dongxu Huang, Qinyu Dang, Lei Bao

**Affiliations:** ^1^ Neurology Department Luhe Hospital Capital Medical University Beijing China; ^2^ School of Public Health Beijing Key Laboratory of Environmental Toxicology Capital Medical University Beijing China; ^3^ Department of Clinical Nutrition Peking University International Hospital Beijing China

**Keywords:** cognitive impairment, inflammatory stress, nuclear factor‐κB, type 2 diabetes mellitus, vitamin D3

## Abstract

Patients with type 2 diabetes mellitus (T2DM) have a higher risk to develop cognitive impairment. Several studies reported the potential roles of vitamin D in prevention of cognitive impairment, but the mechanism remains unclear. The present study aims to investigate the protective effects of vitamin D_3_ on cognitive impairment in db/db mice and to explore the possible mechanism. Twelve‐week‐old male db/db mice were randomly administrated with low, medium, and high dose of vitamin D_3_ (LVD, MVD, and HVD groups, respectively) and equivalent volume vitamin D_3_ solvent (corn oil, DM group) intragastrically. Eight age‐matched db/m mice were given equivalent volume corn oil as normal group. After 16 weeks of vitamin D_3_ treatment, the concentrations of fasting serum glucose in three vitamin D_3_ groups (especially the 1,000 IU/kg·bw dose) were significantly decreased compared with DM group. Pathology revealed that the neuron damage was reduced in vitamin D_3_ groups. MVD intervention significantly shortened the escape latency on day 5 and extended time in the target quadrant. Mice in HVD group had significantly higher exploration time and discrimination index compared with the DM group mice. Moreover, vitamin D_3_ treatment has increased the phosphorylation of cAMP‐response element‐binding protein and the expression of brain‐derived neurotrophic factor and vitamin D receptor. This treatment, meanwhile, has decreased the expression of tumor necrosis factor‐α, the phosphorylation of inhibitor kappa Bα (IκBα), and nuclear factor‐κB p65 (NF‐κB p65) in the hippocampus of db/db mice. These results suggest that vitamin D_3_ alleviated cognitive impairment in the hippocampus of db/db mice. Down‐regulation of the NF‐κB signaling pathway‐related proteins IκBα and p65 might be one of the possible mechanisms.

## Graphical abstract

Our results suggest that vitamin D3 alleviated cognitive impairment in the hippocampus of db/db mice. Down‐regulation of the NF‐κB signaling pathway‐related proteins IκBα and p65 was one of the possible mechanisms.

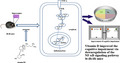

## 1 INTRODUCTION

Type 2 diabetes mellitus (T2DM) is a serious and common clinical chronic disease, which has reached pandemic dimensions all over the world. Cognitive impairment is one of the most frequent diabetic complications. Many studies reported that patients with T2DM showed an increased risk of developing cognitive impairment, and having problems with immediate recall, delayed recall, verbal memory, psychomotor speed, and verbal fluency (Xue et al., 2019). Poor cognitive function may affect the implementation of many diabetes treatment regimens and increase the risk of severe hypoglycemia in T2DM (Punthakee et al., 2012 ^)^. According to the data reported in 2015, the number of older people suffered from dementia accounted for 10.0 million disability‐adjusted life years (DALYs) in 2010, which was forecast to expand by 86% by 2030 (Prince et al., 2015). It was noting that cognitive impairment is the essential stage from the cognitive changes of natural aging to dementia. Thus, it is necessary and imperative to find safe and effective methods to prevent the cognitive impairment in T2DM.

Inflammation is a complex cellular and molecular response to insults (stress, injury, or infection) and as an attempt to defend back (Lee et al., 2010). As a common feature of diabetes and neurodegenerative diseases, such as Alzheimer's disease (AD) and Parkinson's disease, inflammation is playing an important role in the progress of these diseases. The inflammatory responses in neurons, for example, the activation of microglia, macrophages, astrocytes, and lymphocytes, can result in the release of inflammatory mediators, such as chemokines, cytokines, neurotransmitters, and reactive oxygen species (ROS) (Tansey et al., 2007). The release of these mediators brings out the recruitment of lymphocytes and monocytes through the blood–brain barrier (BBB) (Lossinsky & Shivers, 2004; Taupin, 2008). Then, the activation of additional microglia can promote their proliferation, causing further discharge of more inflammatory factors (Das & Basu, 2008). A meta‐analysis study showed that the elderly with AD had the elevated peripheral levels of tumor necrosis factor‐α (TNF‐α), interleukin (IL) ‐6, and IL‐1β (Swardfager et al., 2010). Inflammatory processes are reported to be closely related to β‐cell dysfunction and insulin resistance and to promote the occurrence, progress, and complications of diabetes (Weber et al., 2015). Inflammation may also further impair the cerebral vasoreactivity, leading to a decline in executive function and daily activities performance in older people with T2DM (Chung et al., 2015). Vitamin D is a group of lipid soluble sterols with two major forms: vitamin D_3_ (25‐hydroxvitamin D_3_) and vitamin D_2_ (25‐hydroxvitamin D_2_). Vitamin D_3_ is the most active form of vitamin D after digestion. Vitamin D was reported to play a broad role in cancer, immunity and autoimmune diseases, cardiovascular and respiratory health, diabetes and so on (Christakos et al., 2013). Deficiency and insufficiency of Vitamin D is a worldwide problem which affects more than one billion people (Holick, 2017). A cross‐sectional study, which involved with the middle‐aged and elderly people in Lanzhou China, showed a 75.2% incidence of vitamin D deficiency (25(OH)D levels <20 ng/ml) of the entire study population (*n* = 10,038). Meanwhile, the study indicated that the deficiency was more prevalent in women (79.7%) than in men (64%) (Zhen et al., 2015).

Vitamin D deficiency has been proved to be associated with various neuropsychiatric symptoms, which plays a vital role in the development of dementia. Epidemiologic studies indicated that the serum 25‐hydroxvitamin D level in people with impaired cognitive function and dementia is lower than the level in healthy people (Jamall et al., 2016; Larsson et al., 2018). A prospective pre–postinterventional study found that subjects who received oral vitamin D3 supplementations (800 IU per day or 100,000 IU per month) exhibited improved global cognition and executive functioning abilities compared to controls over a 16‐month follow‐up period (Annweiler et al., 2012). Vitamin D is recognized to have a protective effect on cultured hippocampal cells in vitro against excitotoxic insults. It is probably due to a modulation of L‐type voltage‐sensitive calcium channels, whose increase has been documented in hippocampus aged cells and the aged long‐term cultured hippocampus cells (Gezen‐Ak et al., 2013; Porter et al., 1997). Vitamin D can also regulate nerve growth factor expression, enhance neurite outgrowth, and reduce cellular proliferation in embryonic hippocampal cells (Marini et al., 2012). Additionally, vitamin D binds to its nuclear hormone receptor vitamin D receptor (VDR), which is located in CA1, CA2, CA3 regions, and dentate gyrus of hippocampus. Primary cortical neuron cultures found that mRNA expression levels of VDR and the enzymes involved in bioactivation of vitamin D were higher in hippocampal neurons than the cortical ones, which indicated a “higher requirement of vitamin D” in hippocampus and the potential consequences of vitamin D deficiency in cognitive decline, neurodegeneration, and Alzheimer's disease (Gezen‐Ak et al., 2013). Low vitamin D is proved to have certain association with cognitive deficit. Cross‐Sectional and Longitudinal studies have found that vitamin D levels are significantly low in individuals with cognitive impairment and Alzheimer's disease compared to healthy adults (Beydoun et al., 2018; Jorde et al., 2015). Vitamin D could control the genetic regulation of the synthesis of dopamine (DA), serotonin (5HT3), acetylcholine (Ach), and gamma‐aminobutyric (GABA), which in turn improves neural activity and meliorates symptoms of cognitive impairment (Moretti et al., 2018).

Interestingly, vitamin D deficiency also plays an important role in insulin resistance and the pathogenesis of T2DM. The level of 25(OH)D_3_ was also negatively associated with insulin resistance (Zhang et al., 2016). Additional, vitamin D is reported to have a potential role in chronic inflammation (Oliveira et al., 2017). Although the relationships of vitamin D levels with cognitive impairment in diabetes has been described by several studies, the molecular mechanism of the benefits of vitamin D on cognitive impairment in T2DM remains quite unclear. The aim of the present study is to investigate the potential protective effect of vitamin D_3_ on cognitive impairment in db/db mice and explore the specific mechanisms of inflammation.

## 2 MATERIALS AND METHODS

### 2.1 Materials and reagents

Vitamin D_3_ was purchased from Sigma‐Aldrich Co LLC. (Shanghai, China). All chemicals used in the present study were of analytical grade and purchased from Beijing Chemical Company (Beijing, China) otherwise specified.

### 2.2 Animal experiment

Thirty‐two male C57BLKS/JGpt‐ *Lepr* *^em2Cd^*/Gpt db/db mice and eight age‐matched male db/m mice (12 weeks old) were purchased from Jiangsu GemPharmatech Biological Medicine Co. LTD (Nanjing, China). All the mice were maintained in wire‐topped plastic cages in a 12:12‐hr light–dark cycle and temperature (20 ± 2°C) with ad libitum access to food and water. All animal procedures for this study were approved by the Capital Medical University Animal Experiments and Experimental Animals Management Committee (AEEI‐2018‐136) in accordance with the guidelines of the Chinese Council of Animal Care.

After 1 week of acclimation, the db/db mice were randomly divided into the following four groups (*n* = 8): DM control group, low dose of vitamin D_3_ group (LVD, 250 IU/kg bw), medium dose of vitamin D_3_ group (MVD, 500 IU/kg bw), and high dose of vitamin D_3_ group (HVD, 1,000 IU/kg bw). Corn oil was the solvent of vitamin D_3_. Mice in vitamin D_3_ intervention groups were treated with vitamin D_3_ intragastrically. DM control mice and db/m (used as normal control group) were treated with an equal volume of corn oil. After 16 weeks, body weight and food intake were recorded, and all mice underwent Morris water maze test and Novel object recognition test. The mice were sacrificed by 1% (m/v) pentobarbital (50 mg/kg bw), and blood was collected from carotid artery by vacuum blood collection tubes (BD, *Shanghai*, China) and serum was isolated via centrifugation (3,500 r/min, 4°C, 15 min) and stored at −80°C. The left brains of the three mice randomly selected in each group were quickly removed and fixed in 4% paraformaldehyde for subsequent histological analysis. The right hippocampi of the mice were rapidly dissected and stored at −80°C for Western blots analysis.

### 2.3 Biochemical assay

Fasting serum glucose, high‐sensitivity C‐reactive protein (hs‐CRP), total cholesterol (CHO), triacylglycerol (TG), high‐density lipoprotein cholesterol (HDL‐C), and low‐density lipoprotein cholesterol (LDL‐C) levels were measured by Olympus AU400 automatic biochemistry analyzer (Olympus, Tokyo, Japan). All detection kits were purchased from InTec PRODUCTS, INC. (Xiamen, China).

### 2.4 Histology

After the fixation, the brain samples were embedded in paraffin and then were cut into 5‐μm‐thick coronal sections. Histopathological analysis was carried out using hematoxylin and eosin (HE) and Bielschowsky silver staining. The morphology of the CA1, CA3, and DG regions of hippocampus in each group was scanned by Pannoramic SCAN II (3DHISTECH Ltd., Budapest, Hungary) and captured by CaseViewer software (3DHISTECH Ltd., Budapest, Hungary).

### 2.5 Morris water maze test

The Morris water maze test (MWM) test was performed as described in previous study by Panlab water maze‐smart video tracking from DL NATURGENE LIFE SCIENCE, Inc. (Beijing, China).( Zhang et al., 2015) The water maze consists of a circular pool (120 cm diameter and 60 cm high) filled with 21 ± 1°C water containing white bath additive to a depth of 40 cm, a black escape platform (10 cm diameter and 1 cm below the water level) were located in the middle of the southwest quadrant. Briefly, the pool was divided into 4 quadrants with 4 different color visual cues on the inner wall including triangle, circle, square, and rectangle, respectively. The mice were trained to find the submerged escape platform in water maze for 5 consecutive days, with four trials per day. In each trial, the mice were placed at the starting position of the different quadrants and gently released into the water facing the inner wall and allowed to search the platform for 90 s. The escape platform was removed after 24 hr, and the mice were released from the northeast quadrant and allowed to swim freely for 90 s. All trials were monitored with the SMART v3.0.06 video tracking software, and the performance measurements were extracted including the latency to find the platform (escape latency), times in the target quadrant, distance traveled around the platform, and the number of platform crossings.

### 2.6 Novel object recognition test

Novel object recognition test (NOR) was performed at 16 w. The test is composed of three stages: habituation, familiarization, and probing. Mice were habituated to an opaque arena (50 × 50 × 25 cm) for 10 min on d 1. In the next day, mice were returned to the arena with two identical plastic objects (4 × 4 × 10 cm) for 8 min. On d 3, mice were returned to the arena, in which one of the two familiar objects was replaced by a novel object with the same material but different color and shape. Mice explored the arena for 5 min, and total exploration time was recorded. Exploration was defined as nosing and sniffing at the object from a distance less than 2 cm. The discrimination index was expressed by the ratio (TN−TF)/(TN + TF) [TN = time exploring the novel object, TF = time exploring the familiar object].

### 2.7 Western blot analysis

Total protein was extracted from the frozen hippocampus using RIPA lysis buffer (1% Triton X‐100, 1% deoxycholate, 0.1% SDS) and 1 mM PMSF. After ultrasonication for 5 min, extracts were centrifuged at 12,000 r/min for 15 min at 4°C, and the supernatants were collected. The concentration of total protein in the supernatant was quantified by bicinchoninic acid protein assay. Protein samples (20 μg) were separated by sodium dodecyl sulfate‑polyacrylamide gel electrophoresis on 10% (for β‑actin and phosphorylated‐inhibitor kappa Bα) or 12% (for TNF‐α) gels and then transferred onto 0.45 μm polyvinylidene fluoride membranes (EMD Millipore, Massachusetts, USA). The membranes were blocked with 5% (v/v) nonfat dried milk in Tris‑buffered saline containing 0.05% Tween‑20 (TBS‑T) at room temperature for 1 hr and then incubated with the primary antibody at 4˚C overnight. β‑actin antibody was used at a dilution of 1:2,000, TNF‐α, and phosphorylated‐inhibitor kappa Bα (p‐IκBα) were used at 1:200. Membranes were then washed 3 times in TBS‑T and incubated in HRP‑conjugated secondary antibodies (1:4,000) for 1 hr at 37°C. Protein complexes were detected using enhanced chemiluminescence Western blotting detection reagents (Millipore Corporation, Massachusetts, USA) by FUSION FX EDGE (Vilber Lourmat, Paris, France). Digital images of the blots were analyzed by Fusion‐Evolution Capt software Version 18.00 (Vilber Lourmat, Paris, France).

### 2.8 Capillary Western immunoassay (Wes)

Wes analysis was performed on a Wes system (ProteinSimple, California, USA) using a 12–230 kDa Separation Module (ProteinSimple SM‐W002 or SM‐W004, California, USA) and either the Anti‐Rabbit Detection Module (ProteinSimple DM‐001, California, USA) or the Anti‐Mouse Detection Module (ProteinSimple DM‐002, California, USA), depending on the primary antibody used. Protein extraction was the same as Western blotting. Firstly, protein samples diluted to an appropriate concentration (2 mg/ml or 4 mg/ml) in 0.1x sample buffer and Fluorescent Master Mix were heated at 95°C for 5 min. The samples (3 μL), antibody diluents (10 μL), primary antibodies (10 μL), HRP‐conjugated secondary antibodies (10 μL), and chemiluminescent substrate (15 μL) were pipetted into the plate. The concentrations of samples and antibodies were used as follows: sample concentrations for brain‐derived neurotrophic factor (BDNF) and cAMP‐response element‐binding protein (CREB) were 4 mg/ml; sample concentrations for phosphorylated‐CREB (p‐CREB), vitamin D receptor (VDR), β‑actin, inhibitor kappa Bα (IκBα), nuclear factor‐κB p65 (NF‐κB p65), and phosphorylated‐NF‐κB p65 (p‐NF‐κB p65) were 2 mg/ml. BDNF and β‑actin were diluted at 1:10 and 1:500, respectively; VDR and phosphorylated‐CREB were diluted at 1:20; CREB and phospho‐NF‐κB p65 were diluted at 1:50; IκBα and NF‐κB p65 were diluted at 1:200. The protocol was used as follows: stacking and separation at 375 V for 25 min; antibody diluent for 5 min, primary and secondary antibody both for 30 min; Luminol/peroxide chemiluminescence detection for 15 min. At last, the electropherograms were obtained and analyzed by Compass for SW v4.0.0‐0815.

### 2.9 Statistical analysis

The data are shown as mean ± SE. One‐way analysis of variance was performed using SPSS 22.0 for Windows (SPSS, Inc., Chicago, IL, USA) to compare variances. If variances were equal, Bonferroni multiple comparison tests were performed; otherwise, Tamhane's T2 test was performed by SPSS 22.0. *p* < .05 was considered to indicate a statistically significant difference.

## 3 RESULTS

### 3.1 Effect of vitamin D_3_ on physical and biochemical characteristics of mice

As shown in Table 1, DM mice were heavier than normal mice (*p* < .01). There were no significant weight changes among DM mice and Vitamin D_3_‐treated mice. The concentrations of fasting serum glucose, serum CHO, serum HDL‐c, serum LDL‐c, together with the amount of food intake of DM mice were significantly higher than those of normal mice (*p* < .05 or *p* < .01). After vitamin D_3_ treatment (especially the 1,000 IU/kg bw dose), the levels of fasting serum glucose were significantly decreased (*p* < .05), but no significant alterations were detected in other parameters.

**TABLE 1:** Effect of vitamin D_3_ on physical and biochemical characteristics of mice (*n* = 8)

Variables	Normal	DM	LVD	MVD	HVD
Initial body weight (g)	26.07 ± 0.53	50.20 ± 1.10^**^	48.69 ± 0.84	50.80 ± 1.30	50.84 ± 1.56
Final body weight (g)	29.83 ± 0.68	39.74 ± 2.63^**^	38.37 ± 1.91	42.83 ± 1.83	40.82 ± 1.94
Initial food intake (g/day)	3.03 ± 0.12	6.32 ± 0.25^**^	6.83 ± 0.25	6.56 ± 0.32	7.01 ± 0.43
Final food intake (g/day)	2.66 ± 0.16	7.11 ± 0.52^**^	6.71 ± 0.74	6.37 ± 0.55	5.53 ± 0.82
Fasting serum glucose (mmol/L)	9.38 ± 0.90	22.00 ± 1.51^**^	22.56 ± 1.58	20.21 ± 0.80	18.20 ± 1.38^#^
Fasting serum insulin (μIU/ml)	28.68 ± 5.38	36.72 ± 3.75	37.88 ± 5.12	24.08 ± 3.36	33.79 ± 4.80
Serum hsCRP (mg/L)	0.19 ± 0.04	0.24 ± 0.06	0.23 ± 0.09	0.20 ± 0.02	0.17 ± 0.05
Serum CHO (mmol/L)	2.40 ± 0.47	4.66 ± 0.80^**^	3.71 ± 0.76	3.89 ± 0.22	3.10 ± 0.38
Serum TG (mmol/L)	1.06 ± 0.14	2.23 ± 0.40	2.67 ± 0.91	2.15 ± 0.34	1.86 ± 0.37
Serum HDL‐C (mmol/L)	1.45 ± 0.41	3.07 ± 0.67^*^	2.27 ± 0.61	2.35 ± 0.20	1.89 ± 0.51
Serum LDL‐C (mmol/L)	0.34 ± 0.05	0.60 ± 0.13^*^	0.46 ± 0.11	0.45 ± 0.05	0.38 ± 0.04

Note

Data are expressed as means ± SE.

Abbreviations: CHO, cholesterol; DM, diabetes mellitus; HDL‐C, high‐density lipoprotein cholesterol; hs‐CRP, high‐sensitivity C‐reactive protein; HVD, high‐dose vitamin D_3_ group; LDL‐C, low‐density lipoprotein cholesterol; LVD, low‐dose vitamin D_3_ group; MVD, medium‐dose vitamin D_3_ group; TG, triacylglycerol.

^*^p < .05, ^**^ *p* < .01 versus normal group; ^#^ *p* < .05 versus DM group.

### 3.2 Effect of vitamin D_3_ on the morphology of hippocampus in mice

**FIGURE 1:**
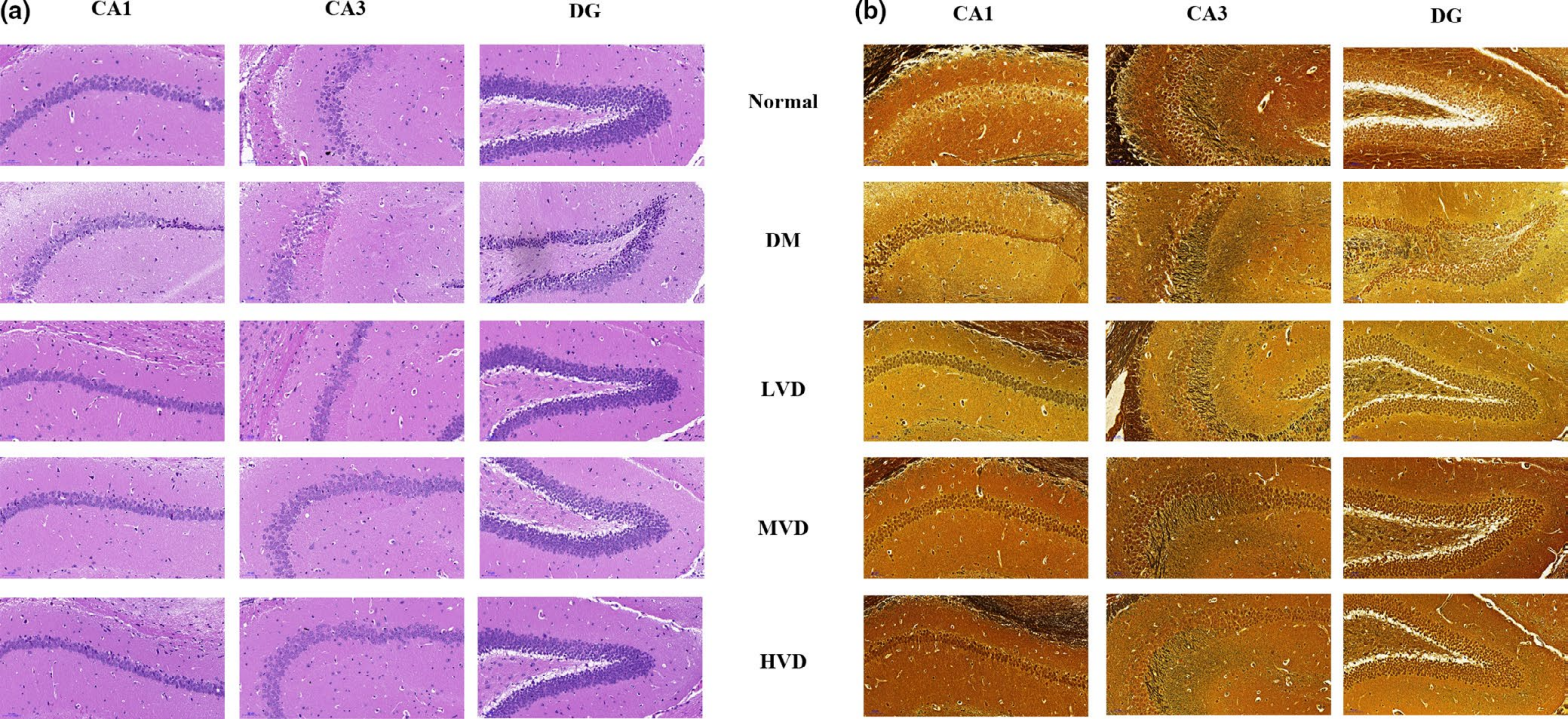
Representative photomicrographs of morphological changes in the hippocampal CA1, CA3 and DG regions (*n* = 3). (a) Representative images of HE stains in the hippocampal CA1, CA3, and DG regions of each group. (b) Representative images of Bielschowsky silver staining in the hippocampal CA1, CA3, and DG regions of each group. HE, hematoxylin and eosin; LVD, low‐dose vitamin D_3_ group; MVD, medium‐dose vitamin D_3_ group; HVD, high‐dose vitamin D_3_ group

We used HE (Figure 1a) and Bielschowsky silver staining (Figure 1b) to observe the morphology changes of the CA1, CA3, and DG regions of hippocampus in each group. Most hippocampal neurons in the normal group were normal and arranged in alignment with obvious nucleolus. In contrast, the morphological structure of hippocampal neurons in DM mice was damaged including the loss of neurons, nucleus shrinkage, edges blur, and neurons were arranged loosely. Notably, neurons in the vitamin D_3_ groups were less damaged and arranged closer compared to the DM group, and the density of pyknotic neurons was markedly decreased than that in the DM group.

### 3.3 Effect of vitamin D_3_ on memory impairment of mice

**FIGURE 2:**
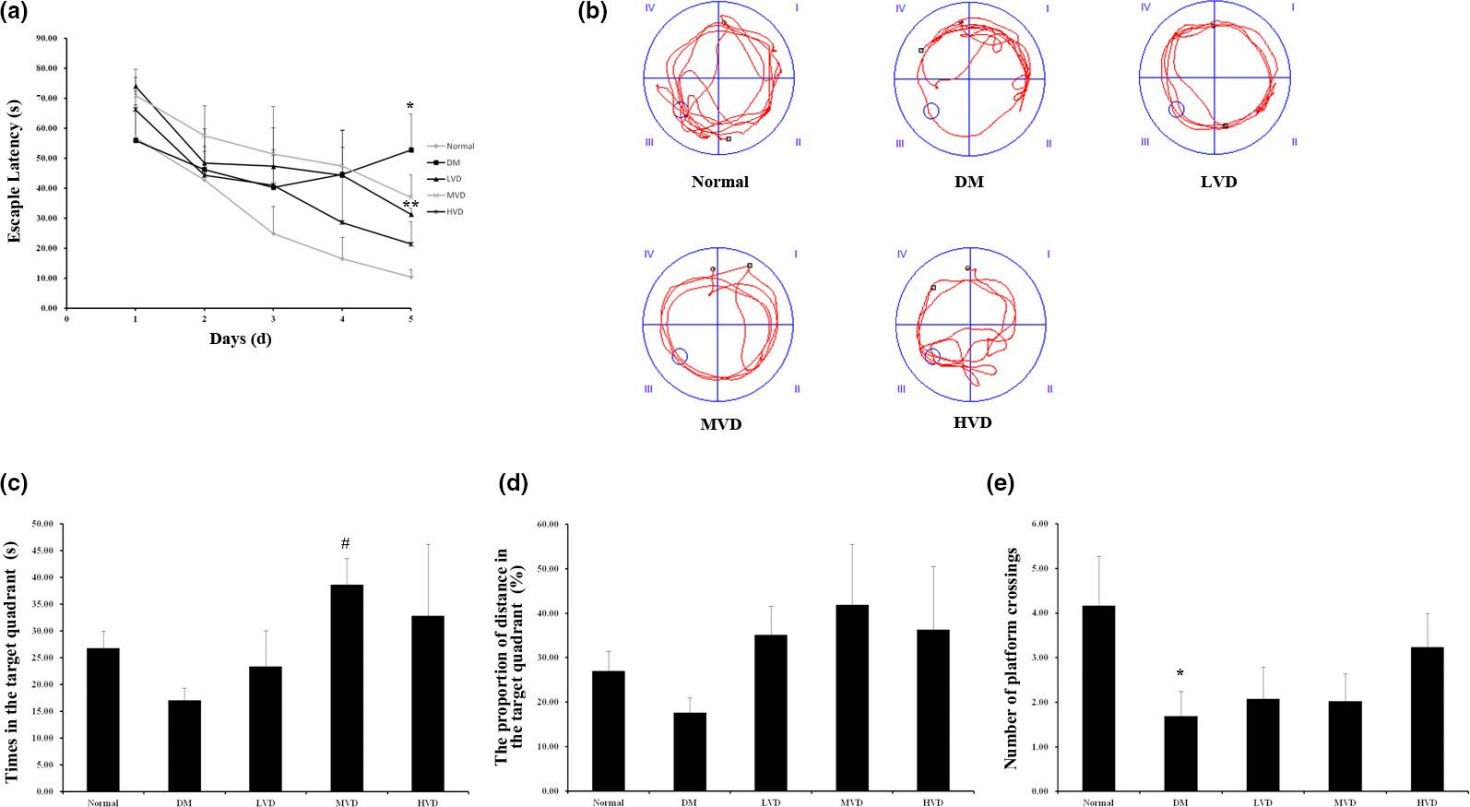
Effect of vitamin D_3_ on memory impairment of mice according to the Morris water maze test (MWM) (*n* = 8). (a) Spatial learning in the MWM on day1 to 5. Average escape latency (s) was shown for the four training sessions in the maze. (b) Representative swimming path of mice in 120 s on day 6. (c) Times in the target quadrant (s) during probe testing on day 6. (d) The proportion of distance in the target quadrant (s) during probe testing. (e) The number of platform crossings. Data were means ± SE. ^*^ *p* < .05, ^**^ *p* < .01 versus normal control rats. ^#^ *p* < .05, versus DM control rats. HVD, high‐dose vitamin D_3_ group; LVD, low‐dose vitamin D_3_ group; MVD, medium‐dose vitamin D_3_ group

The MWM test and the NOR were conducted in this section. The MWM test was performed to elucidate the effect of vitamin D_3_ on hippocampal‐dependent learning and memory of mice. There was no significant difference among groups in terms of the latency to reach the platform (escape latency) on day 1–4. However, the escape latency of DM mice was significantly increased compared with that of normal mice (*p* < .05), and vitamin D_3_ markedly decreased the escape latency on day 5 (*p* < .01) (Figure 2a). In spatial probe testing, times in the target quadrant, the proportion of distance in the target quadrant (s), and the number of platform crossings in DM group were decreased when compared to that in normal group. After the treatment, the 500 IU/kg bw vitamin D_3_ markedly increased times in the target quadrant (*p* < .05). Vitamin D_3_ can also improve the proportion of distance in the target quadrant and the number of platform crossings but no significant differences were found (Figure 2b–e).

**FIGURE 3:**
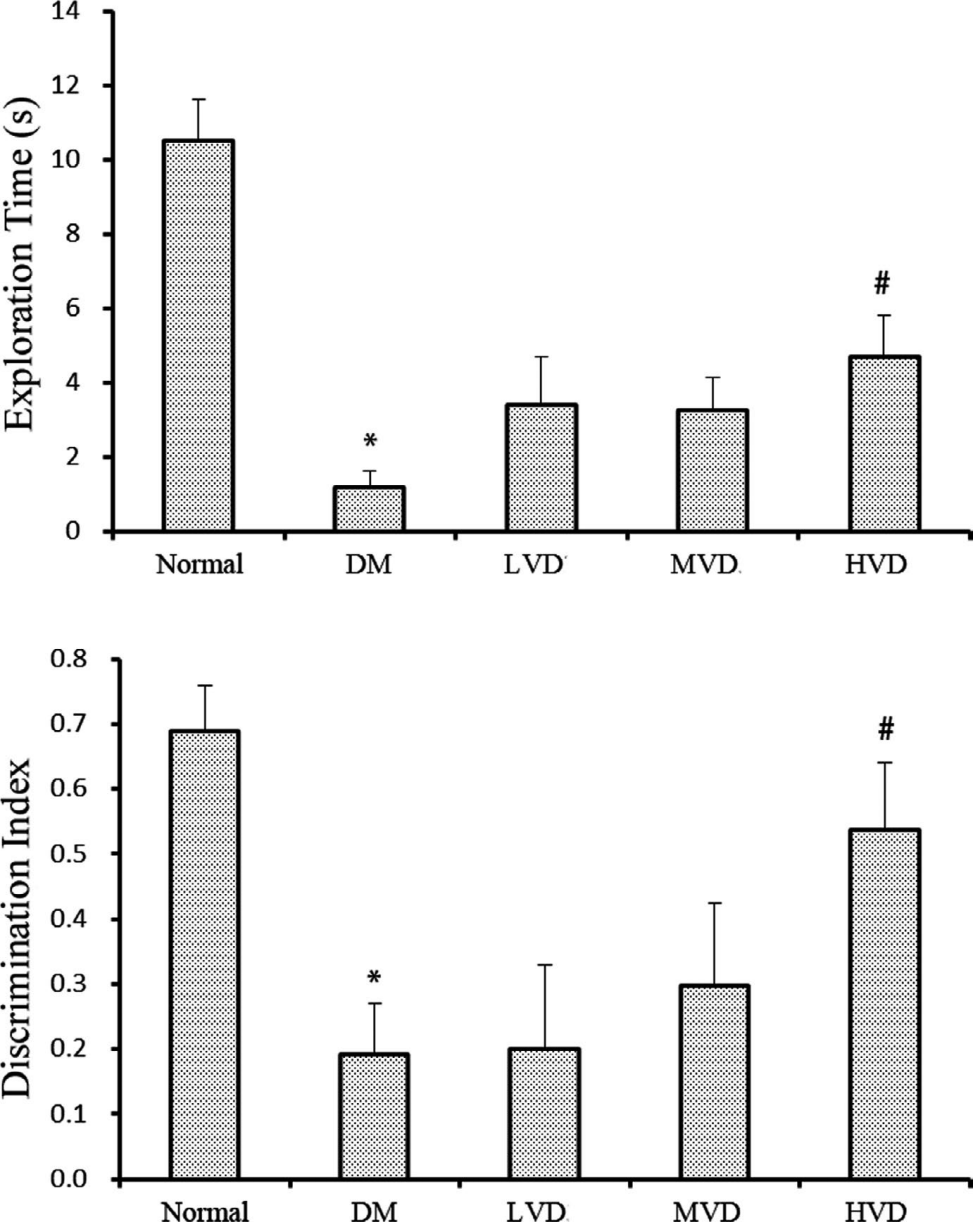
Effect of vitamin D_3_ on the exploration time and discrimination index in the Novel object recognition test (NOR) (*n* = 8). Data were means ± SE. ^*^ *p* < .05, ^**^ *p* < .01 versus normal control rats. ^#^ *p* < .05, versus DM control rats. HVD, high‐dose vitamin D_3_ group; LVD, low‐dose vitamin D_3_ group; MVD, medium‐dose vitamin D_3_ group

The NOR was conducted to examine the recognition memory of mice. As shown in Figure 3, mice in DM group had significantly lower exploration time and discrimination index compared to the normal mice (*p *< .05). Mice in high‐dose vitamin D_3_ group had significantly higher exploration time and discrimination index compared with the DM mice (*p *< .05), which indicated that vitamin D_3_ treatment could improve novel object recognition memory deficit in mice. Collectively, all results above suggest that vitamin D_3_ could improve the memory impairment caused by DM in mice.

### 3.4 >Effect of vitamin D_3_ on the protein expression of VDR, CREB, and BDNF in the hippocampus of mice

**FIGURE 4:**
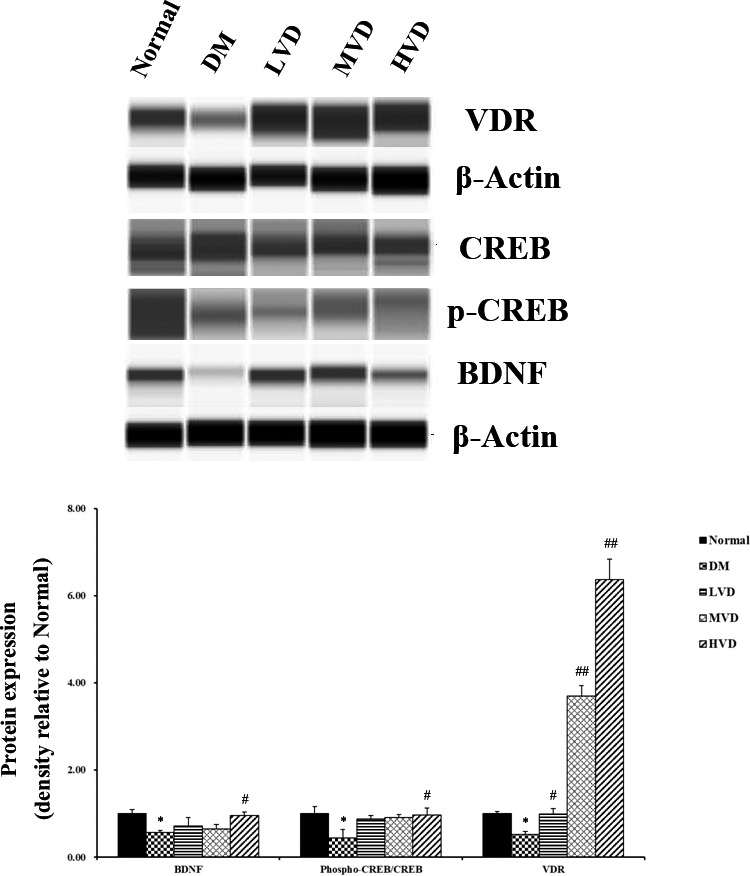
Effect of vitamin D_3_ on the protein expression of VDR, CREB, and BDNF in the hippocampus of mice (*n* = 4). Data were means ± *SD*. ^*^ *p* < .05, versus normal control rats. ^#^ *p* < .05, versus DM control rats. BDNF, brain‐derived neurotrophic factor; p, phosphorylated; CREB, cAMP‐response element‐binding protein; HVD, high‐dose vitamin D3 group; LVD, low‐dose vitamin D_3_ group; MVD, medium‐dose vitamin D_3_ group; VDR, vitamin D receptor

The protein expression of VDR, CREB, and BDNF in the hippocampus of mice was shown in Figure 4. The p‐CREB/CREB ratio and the expression of BDNF and VDR were markedly reduced in DM mice compared with normal mice (*p* < .05 for each). Conversely, treatment with high doses of vitamin D_3_ (1,000 IU/kg bw) significantly improved the p‐CREB/CREB ratio and the expression of BDNF when compared to the DM group (*p* < .05), and the expression of VDR in all vitamin D_3_ groups was significantly increased (*p* < .05 and *p* < .01).

### 3.5 Effect of vitamin D_3_ on inflammatory indicators in the hippocampus of mice

**FIGURE 5:**
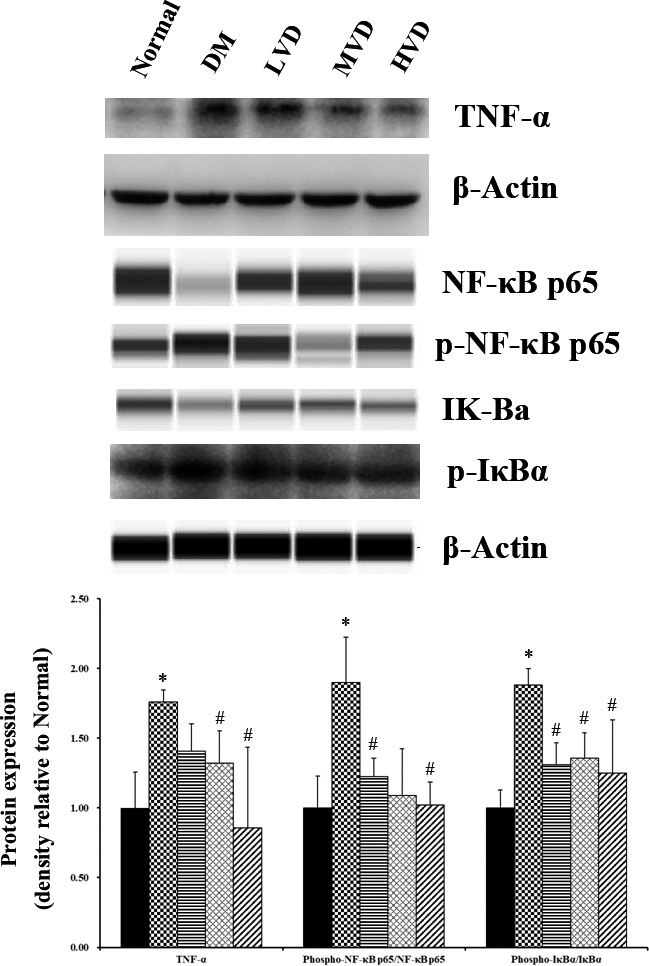
Effect of vitamin D_3_ on the protein expression of TNF‐α, NF‐κB p65, p‐NF‐κB p65, IκBα, and p‐IκBα (*n* = 4). ^*^ *p* < .05, ^**^ *p* < .01 versus normal control rats. ^#^ *p* < .05, versus DM control rats. LVD, low‐dose vitamin D_3_ group; MVD, medium‐dose vitamin D_3_ group; HVD, high‐dose vitamin D_3_ group; TNF‐α, tumor necrosis factor‐α; IκBα, inhibitor of κBα; p, phosphorylated

We evaluated the effect of vitamin D_3_ on inflammation in the hippocampus of mice through detecting the protein expression of TNF‐α, NF‐κB p65, p‐NF‐κB p65, IκBα, and p‐IκBα as can be seen in Figure 5. The expressions of TNF‐α, the p‐NF‐κB p65/NF‐κB p65 ratio, and p‐IκBα/IκBα ratio were markedly increased in DM mice compared with normal mice (*p* < .05), which showed that diabetes significantly exacerbated the inflammatory stress in the hippocampus of mice. Interestingly, vitamin D_3_ treatment reduced the TNF‐α levels, the ratio of p‐NF‐κB p65/NF‐κB p65 and p‐IκBα/IκBα compared with DM mice (*p* < .05).

## 4 DISCUSSION

In the present study, we found that vitamin D_3_ alleviated cognitive impairment in type 2‐diabetic mice. Moreover, vitamin D_3_ decreased the protein expression of TNF‐α, the p‐NF‐κB p65/NF‐κB p65 ratio and p‐IκBα/IκBα ratio in the hippocampus of mice, suggesting that the protective effect of vitamin D_3_ on cognitive impairment caused by diabetes might be related to the inhibition of inflammatory response.

Hyperglycemia is one of the most common symptoms in T2DM and is the leading cause of diabetic complications. It was suggested that the blood glucose fluctuation, insulin resistance, hyperinsulinemia, and long duration of diabetes were the important risk factors of diabetic cognitive impairment (Mortimer et al., 2010). Hyperglycemia could contribute to cognitive disorders, and pathophysiological changes in the brain. Grodstein et al. (2001) observed that diabetic patients had 39% increased risk of Alzheimer's disease and 47% increased risk of dementia compared to the control group. A population‐based study showed that low 25(OH)D levels were correlated with high fasting glucose, insulin resistance, and metabolic syndrome in patients with T2DM and also general population (Oosterwerff et al., 2011). Some clinical trials have been carried out to evaluate the effects of vitamin D_3_ on glucose metabolism, but the results are inconsistent. Nikooyeh et al., (2011) found that the levels of fasting serum glucose, HbA1c, and HOMA‐IR in the vitamin D group were significant lower than those in the control group. A randomized clinical trial (RCT), which was recruiting 50 patients with early T2DM, showed that taking orally vitamin D_3_ supplementation was associated with a transient improvement of fasting plasma glucose after 3 months. However, the effect disappeared after 6 months and there was also no measurable change in β‐cell function (Elkassaby et al., 2014). Another RCT in the United Arabic Emirates showed that there were no effects on fasting blood glucose, C peptide, or HbA1c after taking vitamin D_3_ supplementation for six months in vitamin D‐deficient obese type 2 diabetes patients (Sadiya et al., 2015). Our study showed that vitamin D_3_ decreased the levels of fasting serum glucose in animal model. Our results suggested that vitamin D_3_ has the potential ability to improve abnormal glucose fluctuation. The contradictory conclusions of these studies might be due to multi‐factors, including animal model, sample size, vitamin D dosage, and treatment duration.

It has been reported that low level of serum vitamin D was related to cognitive impairment or dementia of elderly population in several previous studies. A systematic review and meta‐analysis study including 26 studies showed that individuals with low vitamin D status had poorer cognition compared with those with high vitamin D levels (Goodwill & Szoeke, 2017). Chen et al., (2014) found that serum 25(OH)D level was inversely associated with the cognitive impairment in diabetic patients. A prospective cohort study showed that vitamin D deficiency in the short‐term phase of ischemic stroke was associated with a higher incidence of 1‐month cognitive impairment (Chen et al., 2018). In the present study, the results showed the similar trends. Pathology results showed that vitamin D_3_ could decrease the damage of neurons in DM mice. The MWM test showed that vitamin D_3_ could markedly increase extended time in the target quadrant and NOR results indicated that mice in high‐dose vitamin D_3_ group had significantly longer exploration time and higher discrimination index compared with the DM mice respectively. All these results above suggested that vitamin D_3_ could improve the memory impairment in DM mice. Furthermore, we detected the protein expression of VDR, CREB, and BDNF in the hippocampus of mice. VDR is a member of the nuclear receptor super family, which can bind different isotypes of retinoid X receptors (Maestro et al., 2016). The physiological functions of vitamin D are mediated by target genes of the VDR (Carlberg et al., 2012). CREB is one of the best characterized transcription factors which mediates cellular responses in response to various physiological signals such as depolarization, stressors, synaptic activity, neurotransmitters, mitogenic signals, and factors controlling differentiation (Hu et al., 2014). BDNF, the transcription of which is induced by the activation of CREB, is an important neurotrophic factor that participates in various intracellular signaling processes, neuronal protection and survival, morphology of dendritic and axonal cells and synaptic plasticity (Pláteník et al., 2014). In this study, the expression of VDR in all vitamin D_3_ groups was obviously increased, and vitamin D_3_ significantly improved the p‐CREB/CREB ratio and the expression of BDNF. All of the results above suggest that vitamin D_3_ may protect hippocampus against diabetic damage by up‐regulating phosphorylation of CREB and BDNF.

As mentioned above, BDNF is closely correlated with neuronal plasticity, maintenance, survival, and neurotransmitter regulation. Studies have found that BDNF protein levels were reduced both in the brain and serum of patients with psychiatric and neurodegenerative disorders (Autry & Monteggia, 2012; Galvez‐Contreras et al., 2016). These abnormal BDNF levels might be attributable to the chronic inflammatory state of the brain in certain disorders, such as several BDNF‐related signaling pathways are known to be affected by neuroinflammation (Lima et al., 2019). It has been proved that vitamin D possesses anti‐inflammatory property. A cohort study indicated that serum 25(OH)D concentration was inversely associated with several biomarkers of systemic inflammation in extremely obese subjects (Bellia et al., 2013). The similar results were also found in a study performed in older adults (Mellenthin et al., 2014). Furthermore, Mokhtari‐Zaer et al. (2020) found that vitamin D_3_ attenuated lipopolysaccharide‐induced cognitive impairment in rats. In this study, we detected the expression of TNF‐α, NF‐κB p65, p‐NF‐κB p65, IκBα, and p‐IκBα in the hippocampus of mice to evaluate the anti‐inflammatory effect of vitamin D_3_ on diabetic cognitive impairment. TNF‐α is a pro‐inflammatory cytokine and a crucial mediator in tissue damage caused by inflammation (Vielhauer & Mayadas, 2007). An excess of TNF‐α is an important component of the neuroinflammation response related to some neurological disorders (Lawrence, 2009). Our results showed that vitamin D_3_ significantly inhibited the protein expression level of TNF‐α, suggesting that vitamin D_3_ exerted anti‐inflammatory effects on diabetes‐induced cognitive impairment. The nuclear factor NF‐κB is an important regulator that initiates inflammation, and NF‐κB pathway always has been considered to be a prototypical pro‐inflammatory signaling pathway, largely based on the role of NF‐κB in the expression of pro‐inflammatory genes involving cytokines, chemokines, and adhesion molecules (Lawrence, 2009). The NF‐κB signaling pathway is activated by the phosphorylation, ubiquitination, and subsequent proteolytic degradation of NF‐κB‐bound IκB kinase proteins such as IκBα and p65. These proteins in turn trigger the response of cytokine genes such as TNF‐α, IL‐6, and IL‐1β in the nucleus (Kim et al., 2020). Moreover, Bi et al. (2016) reported that venenum bufonis triggers neuroinflammation via activating of NF‐κB pathway, leading to an ultimate decrease in BDNF. Fang et al. (2019) found that neurotropin may reduce memory impairment and neuroinflammation via BDNF/NF‐κB pathway. In agreement, our present study exhibited that treatment with vitamin D_3_ suppressed the phosphorylated forms of p65 and IκBα, which may be a potential mechanism to exert anti‐inflammatory effect on diabetic mice.

In conclusion, our results showed that vitamin D_3_ alleviated cognitive impairment in the hippocampus of db/db mice. Vitamin D_3_ treatment could inhibit inflammatory response which may be attributed to the down‐regulation of the NF‐κB signaling pathway‐related proteins IκBα and p65. Further experimental and clinical studies will be needed to explore the potential protective effects and underlying mechanism of vitamin D in T2DM patients with cognitive impairment.

## CONFLICTS OF INTEREST

The authors declare no conflicts of interest.

## AUTHOR CONTRIBUTIONS

**Xiaomu Tan:** Conceptualization (equal); Funding acquisition (supporting); Project administration (equal); Writing‐original draft (equal). **Lifang Gao:** Data curation (equal); Formal analysis (equal); Methodology (lead); Project administration (lead); Software (equal). **Xiaxia Cai:** Data curation (equal); Funding acquisition (equal); Methodology (equal). **Mingyuan Zhang:** Data curation (equal); Methodology (equal). **Dongxu Huang:** Methodology (equal); Project administration (equal). **Qinyu Dang:** Project administration (equal). **Lei Bao:** Conceptualization (lead); Funding acquisition (equal); Writing‐review & editing (lead).

## Data Availability

The data that support the findings of this study are available from the corresponding author upon reasonable request.
